# Factors Affecting Hatch Success of Hawksbill Sea Turtles on Long Island, Antigua, West Indies

**DOI:** 10.1371/journal.pone.0038472

**Published:** 2012-07-03

**Authors:** Mark Allan Ditmer, Seth Patrick Stapleton

**Affiliations:** 1 Department of Fisheries, Wildlife and Conservation Biology, University of Minnesota, Saint Paul, Minnesota, United States of America; 2 Jumby Bay Hawksbill Project, Jumby Bay, Saint John’s, Antigua, West Indies; Swansea University, United Kingdom

## Abstract

Current understanding of the factors influencing hawksbill sea turtle (*Eretmochelys imbricata*) hatch success is disparate and based on relatively short-term studies or limited sample sizes. Because global populations of hawksbills are heavily depleted, evaluating the parameters that impact hatch success is important to their conservation and recovery. Here, we use data collected by the Jumby Bay Hawksbill Project (JBHP) to investigate hatch success. The JBHP implements saturation tagging protocols to study a hawksbill rookery in Antigua, West Indies. Habitat data, which reflect the varied nesting beaches, are collected at egg deposition, and nest contents are exhumed and categorized post-emergence. We analyzed hatch success using mixed-model analyses with explanatory and predictive datasets. We incorporated a random effect for turtle identity and evaluated environmental, temporal and individual-based reproductive variables. Hatch success averaged 78.6% (SD: 21.2%) during the study period. Highly supported models included multiple covariates, including distance to vegetation, deposition date, individual intra-seasonal nest number, clutch size, organic content, and sand grain size. Nests located in open sand were predicted to produce 10.4 more viable hatchlings per clutch than nests located >1.5 m into vegetation. For an individual first nesting in early July, the fourth nest of the season yielded 13.2 more viable hatchlings than the initial clutch. Generalized beach section and inter-annual variation were also supported in our explanatory dataset, suggesting that gaps remain in our understanding of hatch success. Our findings illustrate that evaluating hatch success is a complex process, involving multiple environmental and individual variables. Although distance to vegetation and hatch success were inversely related, vegetation is an important component of hawksbill nesting habitat, and a more complete assessment of the impacts of specific vegetation types on hatch success and hatchling sex ratios is needed. Future research should explore the roles of sand structure, nest moisture, and local weather conditions.

## Introduction

Globally, hawksbill sea turtle (*Eretmochelys imbricata*) populations have declined by more than 80% from historical levels [1], [2], leading to their designation as critically endangered by the IUCN (2011). Numerous threats and complex life histories, including migrations during different developmental and reproductive stages, complicate management and conservation efforts. While initiatives focused on later sea turtle life stages may have a greater impact on species recovery [3], hatch success is also recognized as an important component for growth and recovery of marine turtle populations in empirical studies [4], [5] and simulations [6]. Additionally, sea turtles (both adult females and their offspring) are most accessible at nesting beaches, thereby providing the opportunity to directly impact their conservation at these life stages. As such, promoting hatch success is often a focus of conservation initiatives. Management agencies and conservation organizations have adopted a number of practices to improve hatch success by replanting native beach vegetation, safeguarding nesting beaches, and relocating nests that are in danger of inundation from tides and rain [7], [8], predation [9], [10], or human-caused disturbances [11]. Better management of vegetation and nesting beaches can further buffer against some impacts of climate change [12], and improved placement of relocated nests may increase the probability of nest success [13].

**Figure 1:**
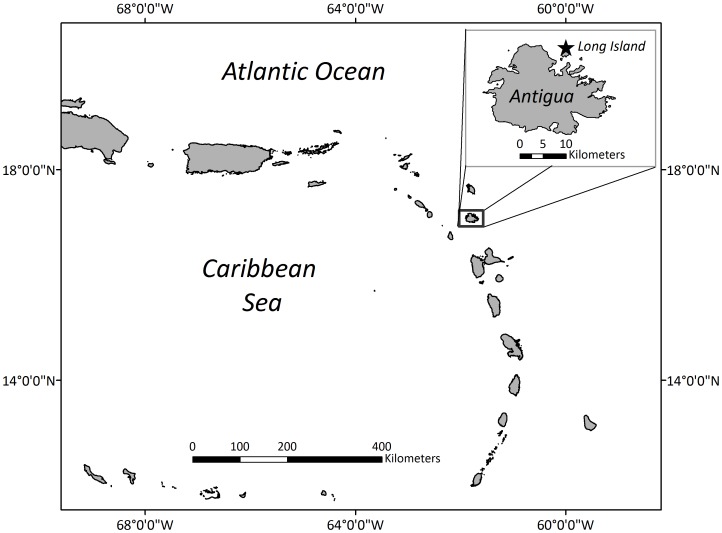
Long Island, or Jumby Bay, Antigua, is located in the Leeward Islands - Eastern Caribbean. GIS data set courtesy of the National Geospatial-Intelligence Agency (2005).

Despite extensive research ranging from the beaches of the United States [14] and the Caribbean [15] to the northern Great Barrier Reef in Australia [16], no consensus has been reached regarding the primary determinants of sea turtle hatch success. Several biological, chemical, physical, and environmental factors have been suggested as possible drivers. Some studies have reported that increased slope of the beach and elevation, which correlates with likelihood of inundation, is linked to higher hatch success [17], [15]; elsewhere, nests deposited closer to the high water mark have greater hatch success [18]. Research has investigated the effects of micro-habitat factors, such as sand characteristics, and found negative impacts on hatch success from increased mean sand grain size, higher levels of sand electrical conductivity, shallow nest depth, large amounts of air-space in the nest cavity, sand type [19] and reduced gas exchange [20]. However, Wallace et al. [21] did not find a relationship between sand characteristics and hatch success. Date of deposition [22] and generalized beach section [16] have also been identified as important predictors of hatch success, but Kamel and Mrosovsky [23] reported no significant relationships between vegetation and hatch success.

Efforts to understand how nesting beach characteristics impact hatch success are complex since hawksbills often exhibit high levels of beach fidelity [24] and nest-site selection within the beach [23]. Moreover, conservation practitioners can use information about the impacts of environmental factors to improve hatch success, but individual fecundity levels also need to be considered when making conclusions about the influence of beach characteristics. There are three major components in measuring an individual sea turtle’s fecundity: the remigration interval, the number of clutches per season, and the number of viable hatchlings per clutch [25]. Little research has investigated causes of individual variation in fecundity, but foraging ground quality [26] and abundance [27], and energy expenditure during nesting attempts [28] are two possible influences. Therefore, a more complete understanding of hatch success not only involves examining nest site selection but also identification of the individual turtle to account for individual fecundity [18], [29].

The Jumby Bay Hawksbill Project (JBHP), initiated in 1987, is a long-term study investigating hawksbill reproductive and nesting ecology. Saturation tagging protocols facilitate the identification of virtually all hawksbills successfully nesting on the study beaches. This unique and expansive dataset affords the opportunity to evaluate individual-specific reproductive parameters, such as reproductive age and how many clutches a hawksbill has previously deposited within that season, in addition to habitat-based metrics.

Our primary objective was to examine variability in hatch success for the Jumby Bay nesting rookery and explore potential environmental, temporal, and reproductive predictors of hatch success. To our knowledge, this represents one of the first attempts to account for variation in individual fecundity while assessing the impact of other parameters on hawksbill hatch success. We additionally use hatch data to create a predictive model that deepens our understanding of nesting ecology and can function as a tool for habitat restoration and better placement of relocated hawksbill nests in similar habitats.

**Table 1:** Covariates included for examining effects of different factors on hatch success of hawksbill sea turtles on Long Island, Antigua, West Indies during the nesting seasons from 2003–2008.

Variable	Description	Mean or Percent of Nests	Range
Temporal Variables			
YEAR	Category for breeding season year (2003–2008)	15%,14%,15%,17%,18%,21%	2003–2008
Julian	Deposition date of the nest	July 30th	June 1st – Sept 20th
Nest#	Observed chronological count for nest of the season per individual(30 day nesting intervals skipped a number)	2.3	1–5
Status	Category for Neophyte or Reimigrant	62%, 38%	–
Environmental/Nest-Site Specific Variables			
BeachSec	7 sections grouped based on broad similarities in environmental features	6%,24%,19%,38%10%,2%,1%	–
Depth	Depth(cm) of nest deposition	47.80	32.0–65.0
VEG	Categories for distance (m) to/from nearest vegetation edge (>1.5 m invegetation, 0.3–1.5 m in vegetation, edge of vegetation, open sand)	46%,23%,16%,15%	–
*HWL	Natural log of distance (m) to mean high tide line	7.1	1.1–27.0
OrgSand	% organic content sand of nest’s grid cell	5.0%	1.7%–7.4%
*LgSand	Square root of % largest sand grain category (>2 mm)	3.3%	0.0%–27.0%
SmSand	Square root of % smallest sand grain category (<.25 mm)	11.1%	0–45%
*ClutchSZ	Square root of the clutch size of the nest	143.7	8–224

* Values listed in untransformed scale.

## Methods

### Ethics Statement

This study was conducted with the consent of the Fisheries Division of Antigua and Barbuda, the permitting and regulatory authority in Antigua and Barbuda. The Jumby Bay Hawksbill Project follows widely accepted best practices for sea turtle research [30]. This project required no animal husbandry and has no permanent direct university affiliation. All research was completed prior to the commencement of the authors’ graduate studies at the University of Minnesota.

**Table 2:** Model selection results from analyses of hatch success of hawksbill sea turtles nesting on Long Island, Antigua, West Indies during the nesting seasons from 2003–2008.

	Model^a^	Parameters (K)^b^	Δ AIC_c_ c	Model Weight^d^
Explanatory Models	Intercept + ID (random intercept) + BeachSec + VEG + Julian^2^+ NestNum^2^+ ClutchSz + Year × Julian + LgSand	27	0.00	0.36
	Intercept + ID (random intercept) + VEG + Julian^2^+ NestNum^2^+ ClutchSz + Year× Julian + LgSand	21	1.39	0.18
	Intercept + ID (random intercept) + BeachSec + VEG + Julian^2^+ NestNum^2^+ ClutchSz + Year × Julian + LgSand + OrgSand	28	1.49	0.17
	Intercept	1	55.62	0.00
Predictive Models	Intercept + ID (random intercept) + VEG + Julian^2^+ NestNum̂2+ ClutchSz+ OrgSand + LgSand	12	0.00	0.28
	Intercept + ID (random intercept) + VEG + Julian^2^+ NestNum^2^+ ClutchSz+ OrgSand + LgSand + Status	13	0.33	0.24
	Intercept + ID (random intercept) + VEG + NestNum^2^+ ClutchSz + OrgSand + LgSand	10	1.16	0.16
	Intercept + ID (random intercept) + VEG + Julian^2^+ NestNum^2^+ ClutchSz+ OrgSand + LgSand + Status + HWL	14	1.87	0.11
	Intercept	1	43.83	0.00

a Explanations for abbreviations can be found in Table 1.

b Number of parameters.

c Change in Akaike’s Information Criterion.

d Relative likelihood of model (i) based on AIC value.

* All other models were more than 2 AICc greater than the best supported model.

Potential covariates in the explanatory model set included all variables listed in Tab 1. The predictive model set did not include categorical terms for nesting-season year (YEAR) and beach section (BeachSec). Models were fit using maximum likelihood and ranked according to differences in Akaike’s information criteria (ΔAIC_c_).

### Study Area

Antigua (17°N, 61°W) is a small island (∼280 km^2^) located in the Leeward Islands of the eastern Caribbean (Figure 1). Long Island, also known as Jumby Bay, is a 120 ha barrier island lying off the northeastern coast of Antigua and serves as the study site of the JBHP. Pasture Bay, a roughly 650 m long, crescent-shaped beach, is the primary nesting site on Long Island. Historically, the calcareous sands of Pasture Bay were abutted by thick maritime forest and coastal shrubs. However, this prime hawksbill nesting habitat has been largely removed for development or destroyed by erosion. To mitigate these losses in nesting habitat, “vegetation islands” of inkberry (*Scaevola sericea*), sea grape (*Coccoloba uvifera*) and other plants were established along central portions of Pasture Bay during the late 1990s.

Numerous smaller beaches flank Pasture Bay and are used for nesting to varying degrees. Nearly all of these peripheral beaches are manmade and adjoin private residences; beaches are wholly or partially nourished with sand, and some are planted with vegetation.

Habitat structure, vegetation type, and sand composition vary dramatically within and across nesting beaches. We classified Pasture Bay and 3 primary peripheral nesting beaches into 7 sections with similar environmental features. The identified beach sections varied in width, distance from the high-water line (HWL) to the edge of the vegetation, degree of disturbance (i.e. proximity to residences and roads, amount of foot traffic), and vegetation types.

**Table 3:** Beta estimates, standard errors and 90% confidence intervals for the covariates included in the top predictive model assessing hawksbill sea turtle hatch success on Long Island, Antigua, West Indies during the nesting seasons from 2003–2008.

Type	Covariates^a^	β	SE(β)	90% Conf. Interval
*Intercept*	Intercept	8.593	4.132	1.797–15.390
*Temporal*	Julian	−0.084	0.039	−0.148– −0.019
	Julian^2^ (quadratic)	0.0002	0.00009	−3.802E^5^– −3.440E^4^
	NEST#	0.788	0.233	0.406–1.171
	NEST#^2^ (quadratic)	−0.110	0.042	−0.179– −0.040
*Environmental*	>1.5 m into VEG	−0.549	0.188	−0.859– −0.239
	1.5 to 0.5 m into VEG	−0.179	0.227	−0.513–0.155
	Open Sand	0.047	0.037	−0.327–0.420
	OrgSand	−11.089	4.931	−19.201– −2.976
	LGSand	1.687	0.538	0.802–2.573
*Clutch Size*	ClutchSz	0.131	0.052	0.045–0.217

a Explanations for abbreviations can be found in Table 1.

The response variable was logit transformed. Reported results were re-fit using restricted maximum likelihood.

**Table 4:** Variable relative importance weights [40] for covariates examined in analyses of hawksbill sea turtle hatch success on Long Island, Antigua, West Indies during the 2003–2008 monitoring seasons.

	Variable Relative Importance Weights	
Variable	Explanatory Models	Predictive Models
Temporal Variables		
YEAR	1.00	NA
Nest#	1.00	1.00
Quadratic: Nest#	0.98	0.93
Julian	0.94	0.93
Interaction: Julian x YEAR	0.93	NA
Quadratic:Julian^2^	0.85	0.82
Status	0.13	0.49
Environmental/Nest-Site Specific Variables		
VEG	1.00	1.00
ClutchSZ	1.00	1.00
LgSand	0.99	1.00
BeachSec	0.67	NA
OrgSand	0.30	0.99
Depth	0.05	0.08
SmSand	0.02	0.02
HWL	<0.01	0.21

Relative importance weights represent the summed weights of all considered models which contain a particular parameter. Covariates are sorted by descending relative weight in the explanatory models.

### Data Collection

We collected data from nesting hawksbill sea turtles during 2003 to 2008. Field seasons extended from June 15^th^ to November 16^th^ during 2003 to 2006 and from June 1^st^ to November 16^th^ in 2007 and 2008. The earlier start to the season in recent years was implemented to accommodate an apparent shift in the peak of the nesting season [31]. The JBHP’s saturation tagging protocols require the commencement of hourly foot patrols in Pasture Bay about 1 hour after sunset each night, with monitoring continuing until the first signs of morning light, shortly before sunrise. Hawksbills require about 1.5 hours to deposit a clutch of eggs, enabling researchers to encounter and identify virtually all successfully nesting turtles. Peripheral beaches were patrolled less regularly and as access permitted, though patrolling intensity increased during greater periods of nesting activity.

**Figure 2:**
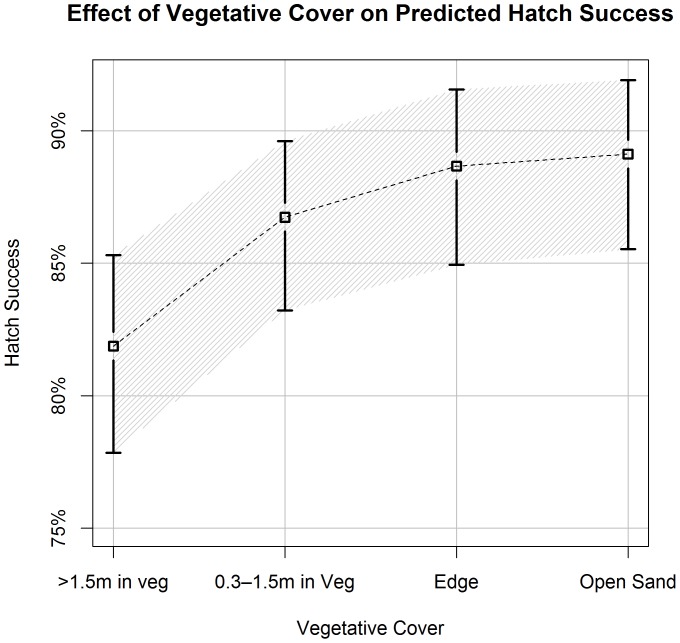
Vegetative cover’s effects on predicted hatch success. Estimates of hawksbill hatch success (±90% CI) in relation to nest vegetative cover in four categories: >1.5 m in vegetation, 0.3–1.5 m in vegetation, 0.3 m in veg to 0.3 m in open sand, >0.3 m in open sand. We derived estimates from the best-approximating model from our predictive model set. All covariates, other than vegetation category, were held constant at their average values (Table 1).

Nesting hawksbills were uniquely marked with metal tags (Inconel no. 681; WIDECAST Marine Turtle Tagging Centre, Barbados) on the trailing edge of both fore flippers during the egg deposition phase to minimize disturbance and the risk of nest abandonment. The supracaudal scutes were similarly marked with a unique combination of holes using a battery-powered hand drill, ensuring that all nesting females remain identifiable throughout their lifetimes. Such identifiers enable the JBHP to track individual reproductive output both within and across seasons. At Jumby Bay, the mean number of hawksbill nests observed per individual is about 4.5 per season, but the modal frequency is around 5 [32]. Hawksbills there maintain a remigration interval (i.e., number of years elapsed between successive nesting seasons) of generally 2 to 4 years [33]. During nesting, distance to the HWL, distance to the nearest vegetation edge, vegetation type, nest site location and several morphometric measurements were recorded. When possible, egg counts were conducted during egg deposition as well.

Hawksbill nests at Jumby Bay hatch about 55 to 70 days after deposition. Nests are typically excavated within 48 hours of emergence to evaluate hatch success and identify potential causes of nest failure. We defined hatch success as the total number of successfully hatched eggs (including hatchlings remaining in the nest; represented by hatched egg shells during nest excavations) divided by the total clutch size, including both hatched and unhatched eggs [34].

During the 2009 season, 30-gram sand samples were collected at a depth of 30 cm from 36 locations across Pasture Beach. Samples were fractionated using mesh sieves with 0.25 mm, 0.5 mm, 1 mm, and 2 mm openings. Following 1 minute of sifting, we weighed the remaining sand by sieve to categorize the percentage of each sample by grain size levels. Sand samples also were burned in a muffle furnace at 500°C for 8 hours to remove organic content. The difference in post-burn weight was divided by the original sample weight to estimate the percentage of organic content within each sample. Each nest was assigned to a sand sample and the corresponding percentage of organic matter and percentage of small (<0.25 mm) and large (>2 mm) grain sizes based on geographic proximity.

**Figure 3:**
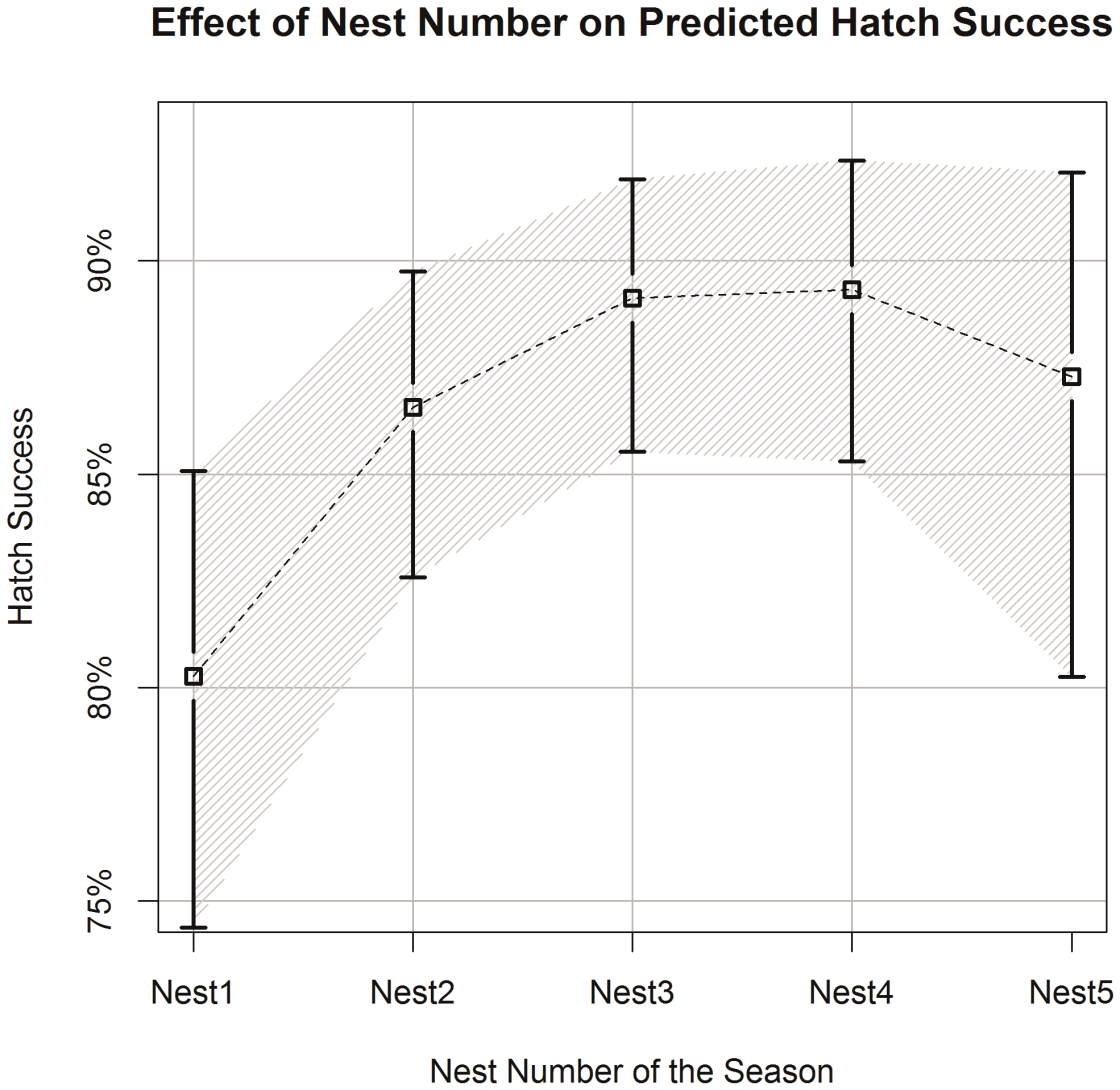
Individual intra-seasonal nest number’s effect on predicted hatch success. Estimates of hawksbill hatch success (±90% CI) in relation to the nest number of the individual turtle within a nesting season. We derived estimates from the best-approximating model from our predictive model set. All numerical covariates, other than nest number (Nest#), were held constant at their average values (Table 1). Open sand was used as the category for vegetative cover for all estimates.

**Figure 4:**
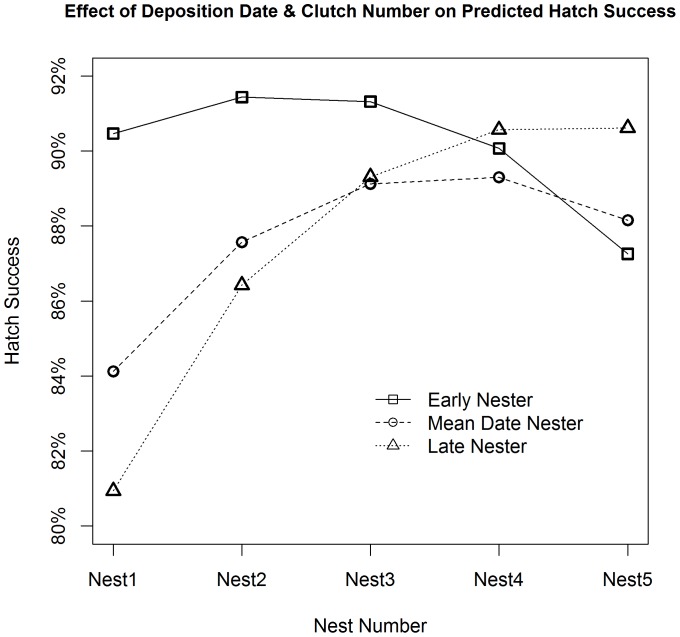
Effects on predicted hatch success from deposition date and individual intra-seasonal nest number. Estimates of hawksbill hatch success in relation to individual intra-seasonal nest number and initial nest deposition dates. Prediction statements used a first nest deposition date of June 1^st^ (date of first monitored nest), July 1^st^ (30 days, 2 15-day nesting intervals prior to mean deposition date), and July 21^st^ (75 days, 5 15-day nesting intervals prior to the maximum observed deposition date) for early, mean date, and late nesters, respectively. All nests were assumed to be deposited at 15-day intervals. Estimates were derived from the best-approximating model from our predictive model set. All numerical covariates, other than nest number (Nest#) and deposition date (Julian), were held constant at their average values (Table 1). Open sand was used as the category for vegetative cover for all estimates.

### Data Analysis

We modeled the logit transformed percentage of hatch success [35] of each nest as a function of environmental, temporal, and breeding history covariates using restricted maximum likelihood with linear mixed models in Program R [36] with package nlme [37]. Nests that were completely washed away due to storms, unable to be relocated for nest excavation, deposited by a turtle that could not be identified or were missing multiple nest-site measurements were not included in this analysis. Nests missing one nest-site measurement were given the mean value of the given variable.

We created two model sets to achieve our study objectives. All possible covariates were included in the global model of an explanatory model set, but a predictive model set excluded categorical variables for the nesting-season year (YEAR) and the broadly delineated section of the beach where the nest was laid (BeachSec). YEAR and BeachSec were included in the explanatory model set to capture any variation that could not be explained by field measurements applicable to other beaches and future nests.

We assessed our two global models using general linear regression. (See Table 1 for a complete list and description of all considered covariates.) We fit several forms of variance structures on our global models using restricted maximum likelihood [38], enabling us to apply likelihood ratio tests to determine if using a random intercept or correlation structure improved fit [39]. We considered random effects’ combinations of intercept and slope for turtle ID, YEAR (factor) and a first order autoregressive correlation structure based on Julian date of nest deposition. We compared the resulting global models for fit using Akaike’s information criteria adjusted for small sample sizes (AIC_c_) [40].

The best fitting global model was then refit using maximum likelihood. We used the re-fit global model and backwards stepwise elimination to remove the least significant covariates (as determined by p-value) until the model was no longer improved from additional removal of covariates [41]. Models were compared by AIC_c_ and model weights (w_i_). After the best-supported model was identified, the data were refit using restricted maximum likelihood to obtain unbiased parameter estimates [39].

**Figure 5:**
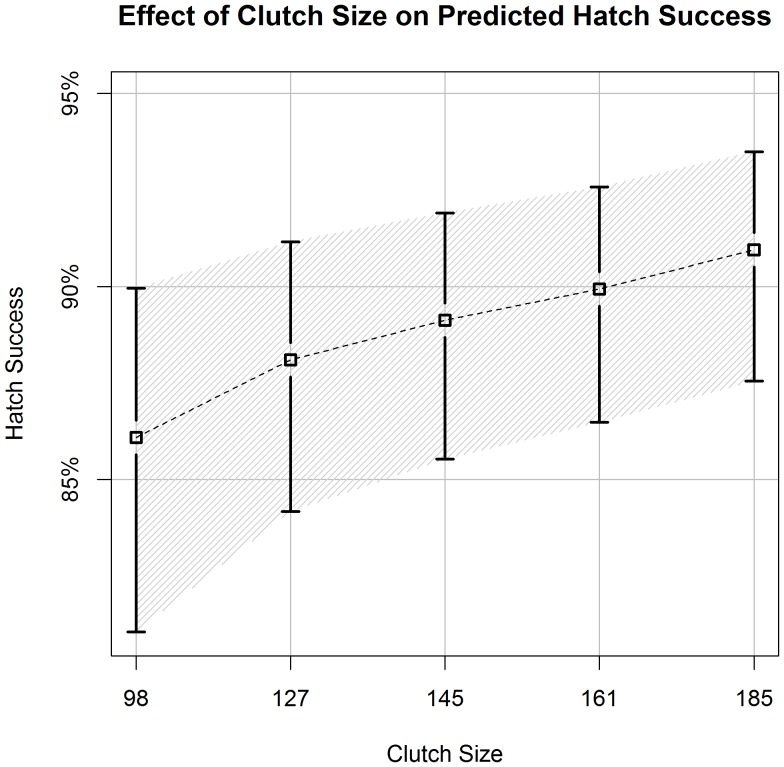
Clutch Size’s Effects on Predicted Hatch Success. Estimates of hawksbill hatch success (±90% CI) in relation to different clutch sizes (quantiles: 5, 25, 50, 75, 95). We derived estimates from the best-approximating model from our predictive model set. All covariates, other than clutch size, were held constant at their average values (Table 1).

**Figure 6:**
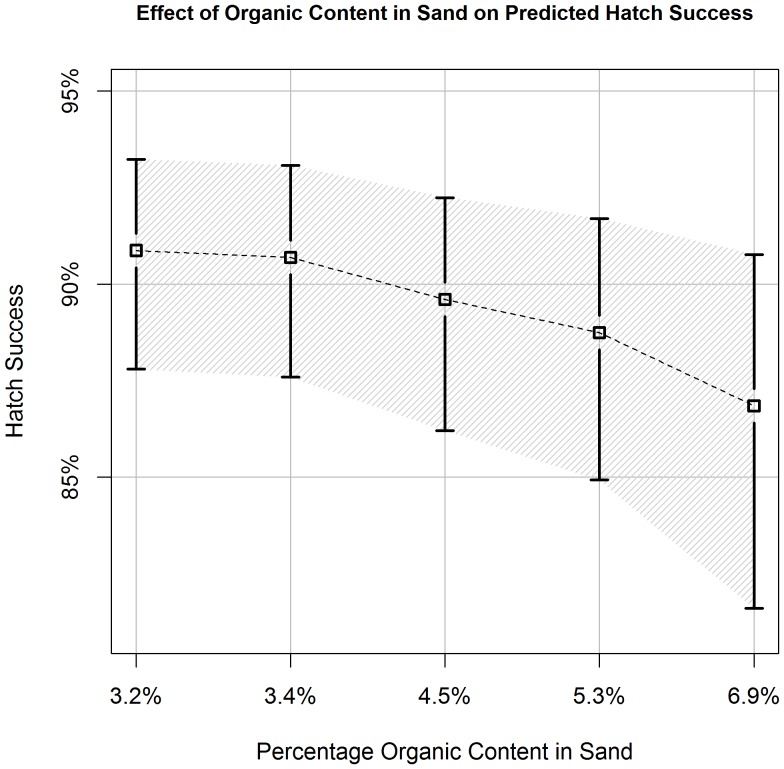
Effect of Organic Content in Sand on Predicted Hatch Success. Estimates of hawksbill hatch success (±90% CI) in relation to different proportions of organic content in sand samples collected across the nesting beach (quantiles: 5, 25, 50, 75, 95). We derived estimates from the best-approximating model from our predictive model set. All covariates, other than clutch size, were held constant at their average values (Table 1).

**Figure 7:**
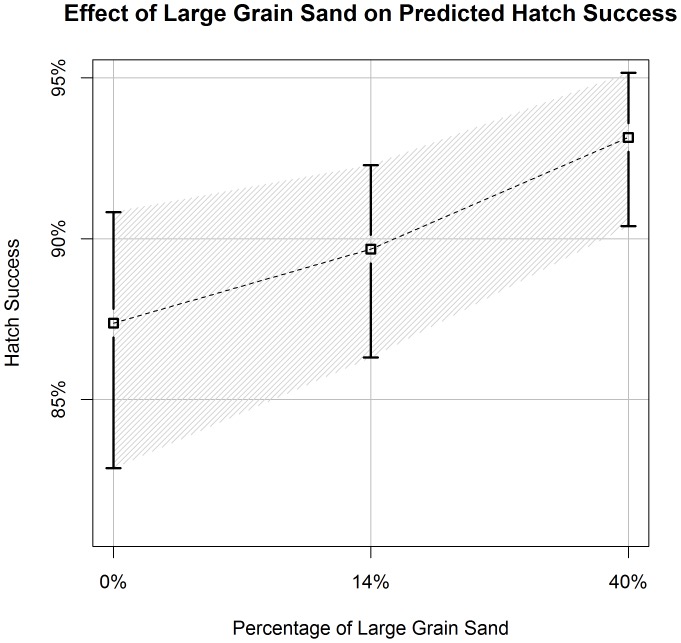
Effect of Large Grain Size Content in Sand on Predicted Hatch Success. Estimates of hawksbill hatch success (±90% CI) in relation to different proportions of large grain size (granules >2 mm) content in sand samples collected across the nesting beach (quantiles: 5, 50, 95). We derived estimates from the best-approximating model from our predictive model set. All covariates, other than clutch size, were held constant at their average values (Table 1).

We back-transformed our parameter estimates to percent of successfully hatched eggs. To present relationships graphically, hatch success and individual covariates were held constant at their mean values, except nest number of the season, for which we used 3 instead of 2.3. We calculated 90% confidence intervals that incorporated prediction uncertainty over all modeled variables by using predict.lme and predictSE.lme [42].

## Results

We analyzed and modeled the hatch success of 652 nests deposited by 198 individual hawksbill sea turtles spanning 6 nesting seasons using 12 temporal, environmental, and reproductive covariates (Table 1). We located and recorded 734 nests but excluded 82 (11.1%) from the analysis because they did not fit criteria for inclusion. The annual number of nests included in analyses ranged from 90 in 2004 to 138 in 2008, averaging 109 nests annually. Hatch success rate varied by 11.8% among years with the lowest rate in 2003 (mean_2003_: 71.7% SD_2003_: 24.4%) and the highest in 2007 (mean_2007_: 83.5% SD_2007_: 16.9%). Mean hatch success across years was 78.6% (SD: 21.2%), and emergence success [35] averaged 76.0% (SD: 22.1%).

Likelihood ratio tests identified the inclusion of a random intercept for individual turtle identity (likelihood ratio test; L  = 20.76, p<0.001). With the complete dataset (explanatory model), AIC_c_ weights indicated that the three best-supported models explained similar amounts of deviance in hatch success and accounted for 72% of the model weight (Table 2). AIC_c_ improved by 55.6 between the null and best-fitting model. By comparison, with the restricted (predictive) dataset, the top three models received 74% of the model weights, with Δ AIC_c_ of 1.87 among them; AIC_c_ improved by 43.8 points between the null and best-fitting models.

Annual variation in hatch success (YEAR) and an interactive term between deposition date (Julian) and nest-season year (YEAR) were included in all three top explanatory models. The top two models in the predictive set included many of the same covariates as the most supported models in the explanatory model set (Table 2). Table 3 reports the beta estimates, standard errors and 90% confidence intervals for the covariates included in the top predictive model. Table 4 provides variable relative importance weights for all models considered.

Each set of models supported the inclusion of a categorical variable for vegetative cover (VEG) and the square root of clutch size (ClutchSz) as well as a quadratic fit for both Julian date (Julian,Julian^2^) and chronological nest number of the season (NEST#,NEST#^2^). The top predictive models included the nest-site specific covariates for % organic content (OrgSand) and % large grain sand (LgSand). Whether the turtle was a neophyte or reimigrant (Status) was supported in two of the top three predictive models and beach section (BeachSec) was supported in two of three explanatory top models.

Vegetative cover (VEG) was the most strongly supported environmental variable in both top model sets: hatch success increased with less vegetative cover (Figure 2). Our best supported predictive model estimates that nests located in open sand average 7.2% higher hatch success than nests with the same characteristics laid more than 1.5 m into vegetation (open sand: 89.1%, 90%CI: 85.5%–91.9% versus >1.5 m in vegetation: 81.9%, 90%CI: 77.6–85.3%; Figure 2). Additionally, of the twenty nests with the lowest hatch success (<20%), 75% (15 nests) were deposited in areas deepest into the vegetation (>1.5%). In contrast, 62% of the nests with the highest hatch success rates (>97%) were located in open sand or edge habitats.

A quadratic effect for the individual turtle’s chronological nest number within season was supported in all top models. The predicted percentage of viable hatchlings increased from the first nest of the season (80.3%, 90%CI: 74.3–85.1%) to the fourth nest (89.3%, 90%CI: 85.3–92.4%) (Figure 3). We also note that, thirteen of the twenty least successful nests (<20% hatch success) were the first nests deposited by individuals within season. However, the temporal effect for date of nest deposition (Julian, Julian^2^) was supported with a negative quadratic fit in both explanatory and predictive models. For a turtle depositing its first nest on July 1^st^ and laying subsequent nests at 15-day intervals thereafter, hatch success differed by about 5% between the first (84.1%, 90%CI: 79.5–87.9%) and fourth nests (89.3%, 90%CI: 85.6–92.1%). Nesting turtles that deposited clutches at the earliest recorded dates in the season had a higher predicted hatch success for the first three nests (90.5%, 91.4%, 91.3%, 90%CI: 84.6–94.3%, 87.4–94.2%, 87.8%–93.9%) compared to individuals beginning to nest on July 21^st^ (80.9%, 86.4%, 89.3%, 90%CI: 75.4–85.4%, 82.3–89.7%,85.8–92.0%) (Figure 4).

Increased clutch size had a positive effect on hatch success. Hatch success for a clutch size of 98 (5^th^quantile) was estimated at 86.1% (90%CI: 81.1–90.0%), while a clutch size of 185 (95^th^ quantile) was 91.0% (90%CI: 87.5–93.5%) (Figure 5). Sand variables for percentage of organic matter and square root of the percentage of large grain sand had opposite effects on nest success in the predictive model (Table 3, Figure 6, Figure 7).

## Discussion

Our results illustrate the complexities of hawksbill sea turtle hatch success and provide an understanding of the role of several environmental and ecological determinants. Our approach, which incorporated saturation tagging to mark individual nesters, allowed us to incorporate a random effect to control for individual variability while assessing the influence of environmental, temporal, and individual nest-level factors. These findings are unique because most previous studies have been unable to assign individual identification to each nest, used nesting beaches where outside forces such as predation [9] or inundation [15], [43] played a major role in hatch success, or were unable to identify specific beach characteristics or seasonal trends that were important drivers of hatch success [16], [44]. We note, however, that Rafferty et al. [29] accounted for individual levels of fecundity while evaluating leatherback (*Dermochelys coriacea*) hatch success and similarly reported that individual identity was an important model component.

Both the explanatory and predictive model sets supported linear terms for vegetative cover and clutch size, and quadratic terms for date of deposition and the individual’s chronological nest of the season. Our top explanatory models also included terms for the nesting season year and a spatial term for the generalized beach section. When these latter variables were excluded, (i.e. our predictive models), the percentages of organic matter and large grain sand were supported in the top models.

Hawksbills are unique among sea turtles in that they tend to nest in or near vegetation; indeed, vegetation is an important factor in hawksbill nest-site selection at our study site [24] and other nesting beaches [45]. Thus, vegetation is considered a critical component of hawksbill nesting habitat. At Jumby Bay, nests located in the deepest vegetation (>1.5 m) were estimated to produce 10.4 fewer viable hatchlings per nest than nests found in open sand. Kamel and Mrosovsky [23], however, did not find a relationship between hatch success and vegetation cover, but they did note a higher emergence success rate and a decreased susceptibility to hatchling disorientation for nests in vegetation compared to nests in open sand. Anecdotally, hatchlings from nests in vegetation appear more susceptible to entanglement in roots at Jumby Bay.

We stress that these findings do not provide a mandate for clearing vegetation from beaches or relocating nests from heavily vegetated sites to more open areas to improve hawksbill hatch success. Our results should be considered in the context of hawksbill ecology, current environmental conditions, a changing climate, and associated changes in sea level, beach disturbance, and temperature. Although our results suggest that increased vegetation is associated with reduced hatch success, vegetation may limit the negative impacts of global climate change, such as erosion from increased sea levels and more powerful storms [46]. Climate change may also affect sea turtle demographics by skewing the sex-ratio towards females in the Caribbean [12]. While Kamel and Mrosovsky [47] suggested vegetation is critical for shading nest sites to help maintain a balanced demography, others have found that vegetation does not significantly influence nest temperature at Jumby Bay [48]. One possible explanation for this contradiction is that different types of vegetation provide different quality of shade and moisture retention. The vegetation on Jumby Bay’s nesting beaches includes native and non-native species and a diverse vegetation structure ranging from beach morning glory (*Ipomoea pes-caprae*) to sea grape and coconut palm (*Cocos nucifera*). We did not assess how differing vegetation types may influence hatch success here. Further research is needed to determine how individual plant species, vegetation structure, and sand albedo [49] influence hatch success as well as nest temperature and hatchling sex ratios.

In some areas, warming sea temperatures have been linked to an earlier onset of sea turtle nesting [50], [51]. In our models, date of nest deposition had a negative quadratic effect on hatch success: nests deposited earlier in the season had higher hatch success. Conversely, all top models supported a term for a strong positive quadratic effect for individual nest number of the season, with hatch success peaking with an individual’s third and fourth nests of the season. For example, our predictive model estimated that an individual depositing her third nest on the population mean deposition date of July 1^st^ produces 13.2 more viable hatchlings in her fourth nest compared to the same individual’s first nest, assuming the same clutch size, location, and deposition date. The quadratic fit suggests a slight reduction in hatch success for the fifth nest of the season, but this reduction was obfuscated by a reduced sample size.

When the effect of deposition date was considered jointly with individual nest number, there was considerable variability in reproductive output between early-season and late-season nesters. Our models estimated that hawksbills that begin nesting around June 1^st^ average 10 more viable hatchlings per nest for their first three clutches than individuals beginning nesting in late July. Research elsewhere has reported reduced hatch success later in the nesting season, but no relationship was found between hatch success and nest number [14]. If we had not included a random effect for turtle identity, results would have only shown a decreasing non-linear trend for deposition date. We acknowledge, however, that nest number and deposition date are somewhat confounded. An individual’s first observed nest was assigned nest number 1 for these analyses, regardless of the deposition date; all early season nests (i.e., within the first 4 weeks of the research season) were therefore categorized as nest numbers 1 or 2. Additionally, some individuals may begin nesting prior to the start of the research season, meaning that although a turtle was depositing her third nest of the year, it was the first nest observed during the research season. We believe that these instances were rare, however, and did not significantly impact our findings.

Our results demonstrated a positive effect of clutch size on hatch success. Other studies have suggested that post-hatch fitness is increased by clutch size due to predator satiation and social facilitation [52], but Mortimer [19] did not report a significant relationship between clutch size and hatch success. We hypothesize that the benefits from increased clutch size for hatch success are likely due to unmeasured parameters at the micro-habitat scale shown to increase hatch success, such as better gas exchange, reduced air nest cavities, and improved temperature regulation or better drainage (reduced impact from inundation) [19], [20]. We note that our results for clutch size showed considerable amounts of variation. The predicted hatch success for a clutch size in the 5^th^ quantile (clutch = 98) has an error estimate (90% CI) that overlaps the error associated with the predicted hatch success of a clutch size in the 95^th^ quantile (clutch = 198).

The support for the term nest year in our explanatory model set suggests our models have room for improvement. In other words, there are likely other factors operating at inter-annual scales that impact hatch success that we have not considered here, such as variation in weather and tidal surges. Hurricane and tropical storm activity data for Antigua are available, but these data alone may not fully represent years with large numbers of inundated nests. Tidal surges from storms can inundate nests, causing both loss of eggs [53] and an inability for researchers to relocate nest positions for excavation. In the future, we hope to more closely monitor weather variables during the nesting season and attempt to quantify the impacts of storms and tidal inundations.

Similarly, the support for the generalized classification of beach section suggests that there are important habitat-related factors influencing hatch success that we have not measured adequately. For example, steep beach slope and increased distance to high tide line have been shown to reduce tidal inundations and improve hatch success [15], but none of our top models supported distance to high tide line. However, this finding was not unexpected, given our exclusion of storm-damaged and missing nests (likely due to erosion and wash-out) from our analysis. Not surprisingly, we anecdotally note that such nests at Jumby Bay were characterized by close proximity to the HWL. Inclusion of both nest year and beach section reduced AIC_c_ 11.8 points between our best explanatory and predictive models.

When beach section was excluded from our predictive models, a negative effect of organic content and a positive effect of large sand grain size (>2 mm) percentage were supported in the top models. Mortimer [19] did not find a relationship between organic content and hatch success of green turtles (*Chelonia mydas)*, but documented a negative relationship between mean sand grain size and clutch survival. She hypothesized that larger grain sand increased the rate of drainage, causing physiological stress to the nest from desiccation. Our measurements for percentage of grain sands ≥2 mm and mean grain size were highly positively correlated (r^2^ = 0.93). We hypothesize that the disparity with our results may be due to climatic differences between Ascension Island and Jumby Bay. We plan to investigate the influence of sand characteristics on hatch success further by analyzing sand collected from individual nest chambers during recent monitoring seasons.

For the colony of hawksbills nesting at Long Island, Antigua, we identified several important factors influencing hatch success. By utilizing linear mixed models we accounted for variable fecundity among individual turtles while explaining spatial and temporal variation in hatch success. This novel approach resulted in support for a negative effect of increased vegetation cover and percentage of organic content in sand, and a negative quadratic effect for date of deposition. Hatch success increased with larger clutch size, a greater amount of large sand grain, and varied based on an individual’s intra-seasonal nest number.

Our results provide insights about how various environmental, temporal, and nest-site specific covariates influence sea turtle hatch success rates. However, further research is needed to develop a more complete understanding of the drivers of hatch success. Additionally, we emphasize that the production of both male and female offspring (i.e., maintenance of sex ratios) is critical to marine turtle conservation and recovery efforts. As such, monitoring incubation temperatures and nest moisture and examining their relationships to vegetation, sand structure and other habitat features, provides a promising and valuable research avenue, since these factors may play an increasingly important role in shaping sea turtle demographics in a changing climate.
